# Effect of Heat Treatment Time and Temperature on the Microstructure and Shape Memory Properties of Nitinol Wires

**DOI:** 10.3390/ma16196480

**Published:** 2023-09-29

**Authors:** Neha Agarwal, Josephine Ryan Murphy, Tina Sadat Hashemi, Theo Mossop, Darragh O’Neill, John Power, Ali Shayegh, Dermot Brabazon

**Affiliations:** 1 School of Mechanical and Manufacturing Engineering, Dublin City University, 9 Dublin, Ireland; josephine.ryanmurphy2@mail.dcu.ie (J.R.M.); tina.hashemi2@mail.dcu.ie (T.S.H.); darragh.oneill86@mail.dcu.ie (D.O.); dermot.brabazon@dcu.ie (D.B.); 2 I-Form Advanced Manufacturing Research Centre, Dublin City University, 9 Dublin, Ireland; theo.mossop@ucdconnect.ie (T.M.); john.power1@ucdconnect.ie (J.P.); ali.shayegh@ucdconnect.ie (A.S.); 3 School of Mechanical and Materials Engineering, University College Dublin, 4 Dublin, Ireland

**Keywords:** nitinol, shape memory effect, heat treatment, precipitates

## Abstract

In this study, the effect of heat treatment parameters on the optimized performance of Ni-rich nickel–titanium wires (NiTi/Nitinol) were investigated that were intended for application as actuators across various industries. In this instance, the maximum recovery strain and actuation angle achievable by a nitinol wire were employed as indicators of optimal performance. Nitinol wires were heat treated at different temperatures, 400–500 °C, and times, 30–120 min, to study the effects of these heat treatment parameters on the actuation performance and properties of the nitinol wires. Assessment covered changes in density, hardness, phase transition temperatures, microstructure, and alloy composition resulting from these heat treatments. DSC analysis revealed a decrease in the austenite transformation temperature, which transitioned from 42.8 °C to 24.39 °C with an increase in heat treatment temperature from 400 °C to 500 °C and was attributed to the formation of Ni_4_Ti_3_ precipitates. Increasing the heat treatment time led to an increase in the austenite transformation temperature. A negative correlation between the hardness of the heat-treated samples and the heat treatment temperature was found. This trend can be attributed to the formation and growth of Ni_4_Ti_3_ precipitates, which in turn affect the matrix properties. A novel approach involving image analysis was utilized as a simple yet robust analysis method for measurement of recovery strain for the wires as they underwent actuation. It was found that increasing heat treatment temperature from 400 °C to 500 °C above 30 min raised recovery strain from 0.001 to 0.01, thereby maximizing the shape memory effect.

## 1. Introduction

**Figure 1:**
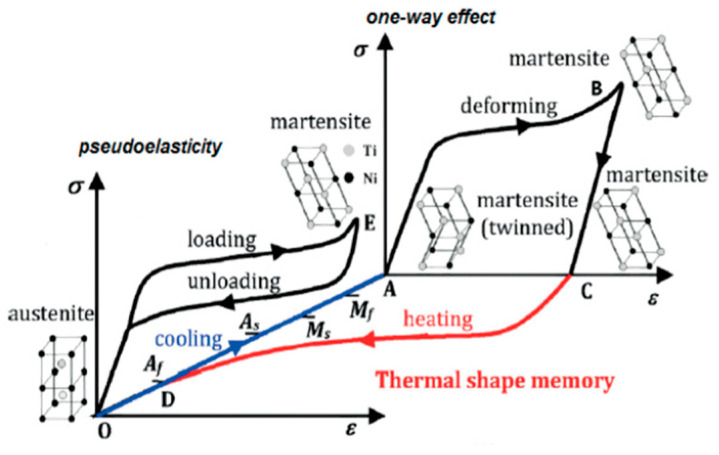
The illustration of the shape memory effect in NiTi alloy is depicted through stress, strain, and temperature variations, adapted from Yuebin Guo et al. [5].

Nitinol is an intermetallic phase of the nickel–titanium alloy with near-equiatomic composition. It has two main crystal phases: a high-temperature austenitic phase with BCC crystal structure, and a low-temperature martensitic phase with a monoclinic lattice structure [1]. The transformation from austenite to twinned martensite on cooling is a diffusionless process meaning that it can occur at very low temperatures as depicted in Figure 1. As there is no diffusion of atoms, the only change that takes place is in the crystal structure of the material, as depicted in Figure 1, allowing what is known as the shape memory effect (SME) to occur [2]. When stress is applied to nitinol in the twinned martensite phase, provided the stress is sufficiently high to induce a phase transformation yet remains below the elastic limit, slip will not take place. Instead, a temporary phase change from twinned martensite to detwinned martensite will be observed [3]. This means that during the phase change, no atomic bonds are broken and thus the phase change is reversible, allowing the nitinol to recover its original shape when heated to the high-temperature austenite phase. The nitinol then remains in this shape as it cools and transforms back to twinned martensite [4].

A process known as shape setting determines the shape that nitinol will return to, the ‘remembered’ shape, once the trained part is heated above the austenite finish (Af) (A_f_) temperature. The most common method of shape setting nitinol is by heat treatment [6,7,8]. The nitinol is held in the desired shape with a mandrel or fixture while it is heat treated at a controlled temperature and time and is then quenched. When the nitinol is removed from the mandrel after quenching, it retains its shape that is the austenitic or “remembered” shape [9]. Another method of shape setting is over-straining that involves cold working nitinol to the point at which full recovery of the previous shape on heating is not possible. This process can be repeated until the previous austenitic shape is sufficiently removed. This procedure is time-consuming and is typically not as repeatable as heat treatment shape setting [10].

The three most important factors in the heat treatment method of shape setting nitinol are the heat treatment temperature, time spent at this temperature, and the raw material condition. Shorter times and lower temperatures result in the material retaining the properties of the high-strength, cold-worked state, but will result in more spring back and less accurate shape setting. Longer times and higher temperatures can result in annealing; however, it also results in shapes that are more exact and have less spring back [11]. The existing literature shows that heat treating nitinol between 400 and 500 °C increases the SME [1,12,13]. This is due to the formation of metastable Ni_4_Ti_3_ precipitates on heat treatment of the samples and also due to the substantial density of reorganized dislocations that remain within the samples [14]. The Ni_4_Ti_3_ precipitates is dominantly present in the {111}_B2_ plane of the NiTi matrix [15]. This is the crystallographic orientation relationship between the Ni_4_Ti_3_ precipitate and the B2 phase. Each of the parent phase has four {111}_B2_ planes with two of these variants having the same plane leading to eight variants of the Ni_4_Ti_3_ precipitates. These precipitates have varying size vastly depending on the heat treatment temperatures and times. The precipitate formation in the ageing temperatures generally have affinity of nucleating and grow near the grain boundaries [16]. It was shown in the work of Sadiq et al. [17] that changes in distribution of the Ni_4_Ti_3_ precipitates governs the SME as it prevents plastic deformation by restricting the movement of dislocations and increasing the critical stress magnitude [17]. From this work, it was also noted that heat treatments at temperatures above 400 °C show the highest extent of the SME. However, in a study by Oncel et al. [7], it was found that, at temperatures above 500 °C, the precipitates are larger, less dense, and begin to re-dissolve within the austenitic phase matrix, decreasing the SME of the material. These studies highlight the importance of finding the optimum heat treatment parameters for nitinol in order to provide the highest extent of SME.

Several research studies have demonstrated that the nickel (Ni) content within NiTi-based alloys and the presence of precipitates, such as NiTi_2_, Ni_3_Ti_2_, and Ni_4_Ti_3_, play a significant role in influencing the alloy’s phase transition temperature. Additionally, the incorporation of alloying elements not only impacts the phase transition temperature but also affects the resultant phase transition products and the pathway of the phase transition [18]. In NiTi binary alloys, a typical one-step phase transition directly from B2 to B19′ is observed. However, the introduction of iron (Fe) results in a two-step phase transition, progressing from B2 to R and then to B19ʹ. Notably, the degree of separation between these two transition steps becomes more pronounced with higher levels of Fe incorporation. The NiTi-Cu alloy system offers less sensitivity of the alloy’s phase transition temperature due to compositional variations, facilitating enhanced control over memory performance. The addition of copper (Cu) imparts the alloy with a high phase transition temperature, exceeding room temperature, enabling the realization of shape memory effects even at ambient conditions. These favorable attributes position NiTi-Cu alloy as a highly promising choice for practical applications in shape memory alloys. Additionally, incorporating palladium (Pd) into a NiTi alloy can substitute for nickel (Ni) atoms, resulting in an initial decrease in the alloy’s phase transition temperature. However, as the quantity of added Pd increases, the phase transition temperature gradually rises with the Pd content [19]. The critical influence of nickel (Ni) content and precipitates on the phase transition temperature of NiTi-based alloys, as well as the profound impact of alloying elements on both the temperature and the characteristics of phase transitions, offers opportunities for tailoring shape memory alloys to suit various practical applications.

Many different methods have been presented in the literature to determine the efficacy of heat treatment parameters on the SME of nitinol. In a study conducted by Lahoz and Puertolas [20], NiTi wire with A_f_ temperature of 29.2 °C was heated treated at 660 °C for 30 min and then quenched in water. Stress–strain and deformation–temperature curves of the NiTi wire were then obtained using a thermomechanical analyzer (TMA). Several different studies used a low-friction linear variable displacement transducer (LVDT) to measure the displacement of a nitinol part. In Lou and Abel [21], NiTi wire with an A_f_ temperature of 80 °C, heat treated at 550 °C for 20 min, was held stationary at one end by a support structure, while the other end was attached to the central slider of a LVDT that measured the horizontal displacement produced by SME. In Speicher et al. [22], nitinol helical springs and Belleville washers are tested using a cylindrical damper as a tension/compression device, and a LVDT was used to measure the vertical displacement during actuation of the nitinol component.

In recent years, there has been a growing interest in harnessing the potential of shape memory alloys (SMAs) like nitinol to enhance structural behaviors. Nitinol, with its remarkable tensile strain recovery properties, offers the ability to generate and withstand substantial stresses and strains, making it a standout candidate for applications requiring high mechanical energy density. This feature enables more compact structural designs compared to other actuator systems and adaptive materials. Furthermore, nitinol’s attributes, such as its robust tensile strength, corrosion resistance, and biocompatibility, have solidified its popularity in various fields, particularly in biomedical applications [23].

Moreover, nitinol-based single-stage bellows, shaped using the rubber bulge method, have been investigated for their compressive behavior. These structures hold potential in energy absorption applications, providing adjustability in energy absorption and crushing forces based on their bellows’ shapes. Additionally, nitinol-based bellows can be reused due to their recovery function, making them a promising choice for energy absorption devices [24,25].

In a separate development, researchers have introduced a novel class of superelastic NiTi honeycomb structures. A unique brazing technique was employed to create nitinol-based cellular structures with low relative densities. These structures exhibited impressive specific strength, high specific stiffness, and enhanced shape recovery when subjected to compression loading, outperforming monolithic shape memory alloys. This breakthrough opens the door to a wide range of engineered topologies with customizable properties and enhanced thermomechanical response [26]. These applications underscore the versatility and promise of nitinol in various fields, from seismic engineering to biomedical devices and advanced structural materials.

While these studies also investigate the effect of heat treatment parameters on the SME of nitinol, there are still several gaps in our understanding of the process and material behavior. Achieving optimal energy consumption and SME performance for NiTi involves selecting a temperature that closely aligns with the operational environment, avoiding being too close to prevent unintended actuation. Previous investigations have shown that the initial A_f_ temperature of the wires were above room temperature; the A_f_ temperature further increased after heat treatments. There are, however, many potential applications for NiTi closer to room temperature so by selecting NiTi with a starting A_f_ slightly below room temperature, after heat treatment, it becomes possible to establish the resulting Af temperature at an appropriate level above room temperature. Studies to date have also only examined a very limited range of heat treatment times and temperatures [27]. Finally, mechanical device methods of displacement and strain measurement used in past studies are known to have errors due to inherent friction and strain accommodation within the connecting mechanical elements [28]. The study involved an extensive exploration of various temperatures and durations applied to nitinol wire with initial A_f_ temperatures below room temperature. The purpose was to determine the most effective heat treatment parameters to enhance shape memory effect (SME) control. Additionally, a strain recovery measurement was conducted using an image analysis methodology.

## 2. Materials and Methods

**Figure 2:**
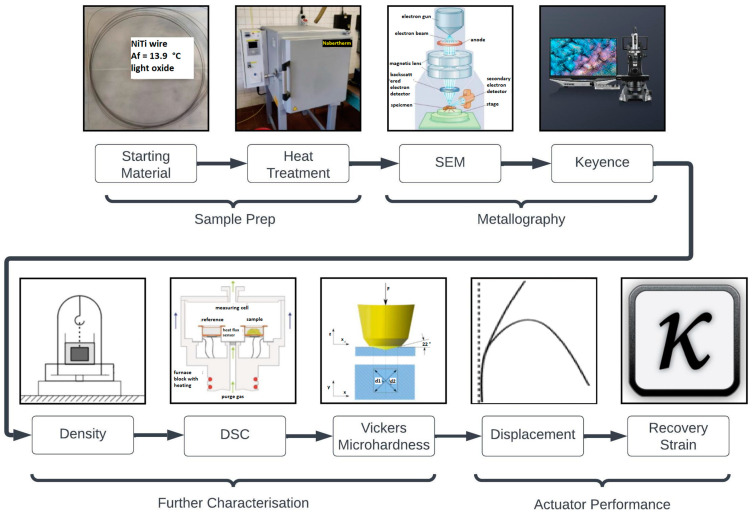
Process diagram showing the implemented experimental procedure.

In this study, a DoE with two parameters at three levels was used to heat treat the nitinol wires in order to determine the optimum heat treatment parameters for maximum shape memory response. Wire samples of length 400 mm were heat treated and shape set as straight wires in a box furnace for 30, 60, and 90 min at 400 °C, 450 °C, and 500 °C. For each of these parameters, two samples were tested in order to validate results and calculate the repeatability. These samples underwent characterization through metallography, density, hardness, and DSC measurements. These analyses were performed to investigate the influence of the heat treatment parameters on the material properties, as depicted in Figure 2. Finally, the shape memory effect (SME) of the samples was tested by angular displacement and recovery strain experiments to determine the optimum heat treatment parameters for SME in nitinol wires. Note that throughout this study *n* = 2 and error bars shown are 95% confidence intervals.

### 2.1. Heat Treatment of Nitinol Wires

The nitinol (NiTi) wire used for this study was provided by Fort Wayne Metals, Castlebar, Co. Mayo, Ireland, with a chemical composition of 55 wt.% Ni and 45 wt.% Ti. The wire supplied had a diameter of 0.468 mm with austenite finish temperature (A_f_) of 13.7 °C with a lightly oxide surface finish. For heat treating the wires, a Nabertherm N60/85HA chamber furnace was used. This furnace has an operating temperature range of 30 to 850 °C and its inner chamber’s dimensions are 350 × 500 × 350 mm. This furnace is heated from the bottom, sides, and the top and uses recirculating air flow to maintain temperature uniformity within 10 K according to DIN 17052-1 [29].

A summary of the examined heat treatment temperatures and times is shown in Table 1. For each specific time and temperature configuration, a 150 mm wire length was subjected to the heat treatment process. In total, nine wires were heat treated in this study. All the samples were heat treated individually to have stability at the respective temperature, thereby avoiding opening of the furnace to add/remove samples. The samples were air cooled outside the furnace upon completion of each heat treatment process.

### 2.2. Metallography

Metallographic analysis of the surface morphology and topology was conducted using a SEM (Zeiss EVO LS15) at an accelerating voltage of 20 kV. The Zeiss EVO LS15 is a thermionic emission SEM with variable pressure option, and a maximum magnification of 300,000× with a 5-axis stage. The thin wires were mounted on conductive stubs and placed inside the SEM chamber for analysis. As the NiTi wires are conductive, no additional sample preparation was required. An Oxford Instrument EDX (Energy Dispersive X-ray) was used for composition analysis. This analysis helped in identifying change in the Ni content as an effect of the variation in the heat treatment process parameters. As titanium is a highly oxidizing element [30], in order to study the microstructure, samples were etched with a Kroll’s reagent (HFbased acid) to provide a better contrast between chemically different features of the material. Nitinol is a particularly unreactive metal in part due to the formation of an insoluble titanium oxide layer. Unlike with primarily titanium-based alloys, this etching procedure is particularly sensitive to alloy segregation, as the chemical reactivity of nickel is significantly different from that of titanium. This creates a challenge for determining the etching time for this metal. The etching times were found to be different for the as-received and heat-treated samples. The etching times used for the samples ranged between 2–5 s. There were no significant differences observed in the microstructures after etching.

### 2.3. Density

The most widely used method for calculating the density of solids is the buoyancy method, which is based on the Archimedean principle. This principle states that a body immersed in a fluid indicates an apparent loss in weight equal to the weight of the fluid it displaces. Ethanol was used as the fluid medium for density measurement in this study [31]. Density measurements were conducted on the nitinol wires, which were heat treated. Three measurements for each sample were recorded with averages and confidence intervals reported in this study.

### 2.4. Differential Scanning Calorimetry

Differential Scanning Calorimetric analysis was performed using a TA Instruments^®^ Discovery DSC2500, New Castle, DE, USA. DSC analysis was performed on all the heat-treated samples as well on the as-received wire to study the phase transformation temperatures. The crucibles used for this experiment were made of pure aluminum, which is suitable for solid and powder samples that do not decompose or boil at temperatures between −170 °C to 600 °C [32]. The tests were conducted in the temperature range of 0 °C to 150 °C with a ramp up rate of 10 °C/min. The austenite and martensite start and peak and finish temperatures were evaluated. TZero aluminum pan with TZero lids and a sample mass of around 20 mg were used to carry out the tests. Liquid nitrogen was used as the cooling medium.

### 2.5. Vickers Microhardness

Vickers microhardness was recorded using a Leitz MiniLoad 2^®^ equipment (Spectrographiv, Leeds, UK) by applying a force of 981 mN onto the surface of the samples with a holding time of 20 s [33]. Vickers hardness measurements were performed on all heat-treated samples, with each sample undergoing five measurements at randomly selected locations on its surface. The resulting average Vickers hardness values were then recorded and reported. For ease of the microhardness measurements with these thin wires, the wires were mounted in epoxy resin and grinded till the required surface finish was attained.

### 2.6. Displacement Experiment of Heat-Treated Nitinol Wires

**Figure 3:**
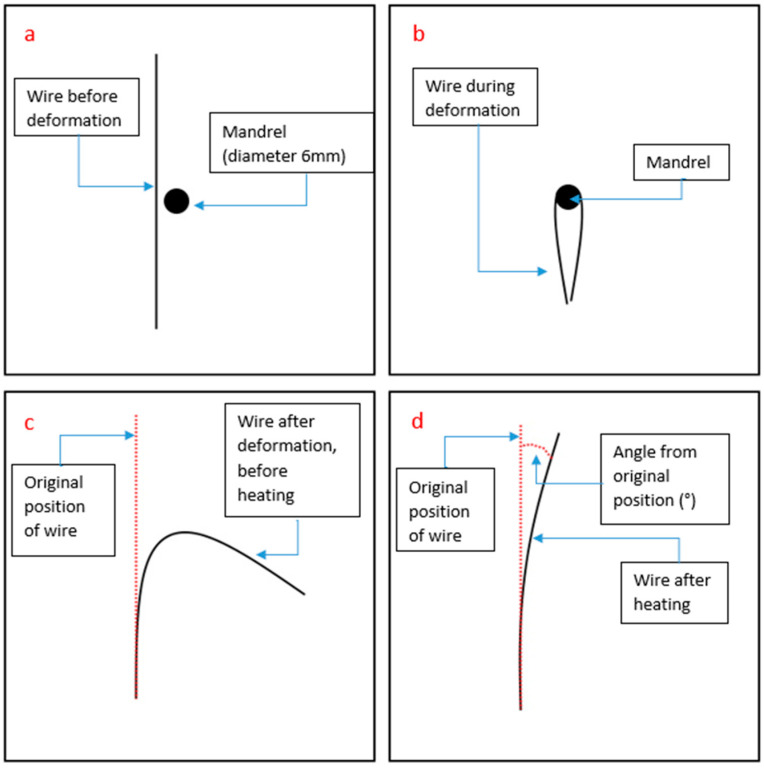
Schematic of the method used for the displacement and recovery strain experiments. (**a**) Wire and mandrel before deformation, (**b**) deformation of wire around mandrel, (**c**) wire after it has been released from the mandrel, and (**d**) wire after controlled heating.

The 150 mm long heat-treated wires were deformed into ‘U’ shapes by bending the wires 180° around a 6 mm diameter mandrel while both the ends were held together. This bending process was carried out with careful attention to prevent kinks in the wire. Such kinks could potentially cause a form of irreversible deformation due to elevated internal stresses compared to the surrounding wire. This deformation, if present, might diminish the overall shape memory effect. The wire was then released and secured to a reference sheet. A controlled heating was then applied to raise the temperature of the wire above the austenite finish temperature. A photograph of the wire, normal to the surface, of each sample after heating was taken, and for the measurement of deformation, an image analysis utilizing ImageJ software (V 1.8.0) was employed [34]. A schematic representation of this entire process is visually outlined in Figure 3.

### 2.7. Recovery Strain of Heat-Treated Nitinol Wires

Images were captured before and after bending for all of the heat-treated nitinol wires. ImageJ software was used to calculate the radius of curvature before and after the wires were heated above the A_f_ temperature. The radius of curvature was calculated using a developed ImageJ macro. Utilizing the initial radius of curvature (denoted by *R*), the bending stress equation was used to find the strain (denoted by ε) in the maximum bent section of the wire. The stress (denoted by σ) over the point of maximum stress equals the Young’s modulus (denoted by *E*) divided by the radius of curvature as in Equation (1).

(1) $$\frac{\sigma }{y}=\frac{E}{R}$$

Furthermore, it is known that strain equals the Young’s modulus by the stress as shown in Equation (2).

(2) σ=E.ε

The combination of these two equations suggests that the strain within the sections of wire that is measured equals the point of maximum stress by the radius of curvature as shown in Equation (3). The point of maximum stress is relative to the neutral axis. The neutral axis within the wires is the center point in the wires. The point of maximum stress is equal to the radius of these wires. Strain was calculated before and after the wires were exposed to the heat source. Recovery strain was calculated by subtracting the strain after recovery from the strain measured before recovery.

(3) $$\varepsilon =\frac{y}{R}$$

## 3. Results

### 3.1. Effect of Heat Treatment on Density

**Figure 4:**
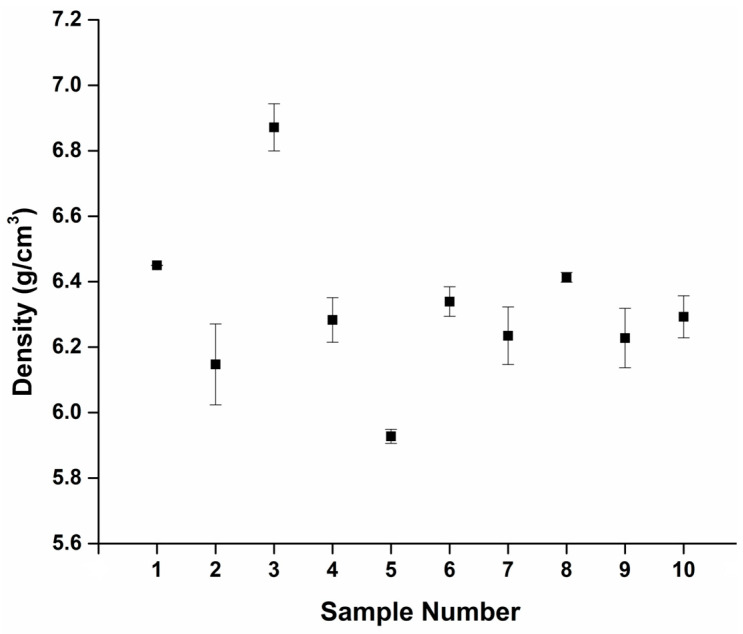
Density measurements (black squares) of all heat-treated nitinol samples, *n* = 3.

Density measurements are conducted to evaluate the impact of heat treatment and its duration on the material’s properties and characteristics. This allowed a comprehensive understanding of how changes in heat treatment conditions affect density and, by extension, the alloy’s performance. While the density of the as-received nitinol was found to be close to the expected nitinol density of 6.45 g/cm^3^ [35], differences in the resultant density were recorded for samples produced at the different processing parameters, see Figure 4. A slight decrease in density compared to the non-heat-treated samples was observed in the samples heat treated at 400 °C for 30 min (sample 2). Among the various heat treatment conditions, the sample treated at 400 °C for 60 min (sample 3) exhibited the highest density, with a value of 6.85 g/cm^3^, while the sample subjected to heat treatment at 450 °C for 30 min (sample 5) demonstrated the lowest density of 5.95 g/cm^3^. A decrease in density relative to the non-heated samples was observed for samples processed at all other heat treatment conditions. The density for the other heat treatment conditions was on average 6.25 g/cm^3^. The density for the sample heat treated at 500 °C for 30 min (sample 8) was the closest to that of the as-received nitinol wire. Both samples, as received and heat treated, were also found to have the lowest standard deviation. It was observed that the heat treatment time had relatively little effect on the density value at 500 °C. Apart from one heat treatment condition, the heat treatments led to a decrease in the density relative to that of the as-received nitinol wire, which is consistent with previous research findings [36].

### 3.2. Effect of Heat Treatment on Microstructure

**Figure 5:**
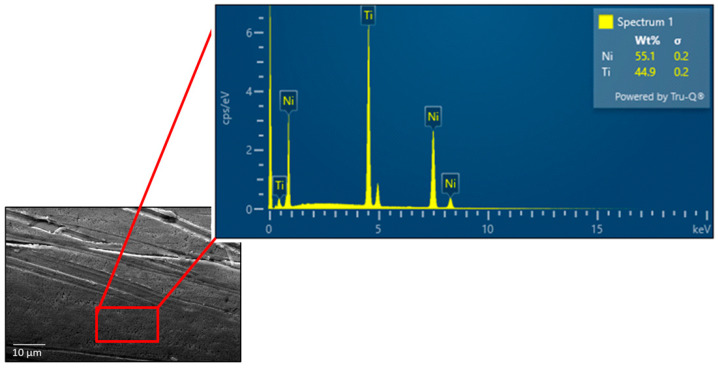
SEM image and EDX mapping of the surface of the as-received wire.

The EDS results of the as-received wire showed a uniform distribution of Ni and Ti. Results of further EDS tests at different locations and areas confirmed that the surface of these samples contained a uniform composition of about 55 wt.% Ni and 45 wt.% Ti as shown in Figure 5. The composition was found to be very close to that reported by the manufacturer. The chemical composition of the heat treated wires has been tabulated in Table 2.

Other observed elements were C and O but were insignificant to be mentioned.

### 3.3. Effect of Heat Treatment on the Phase Transformation Temperatures

**Figure 6:**
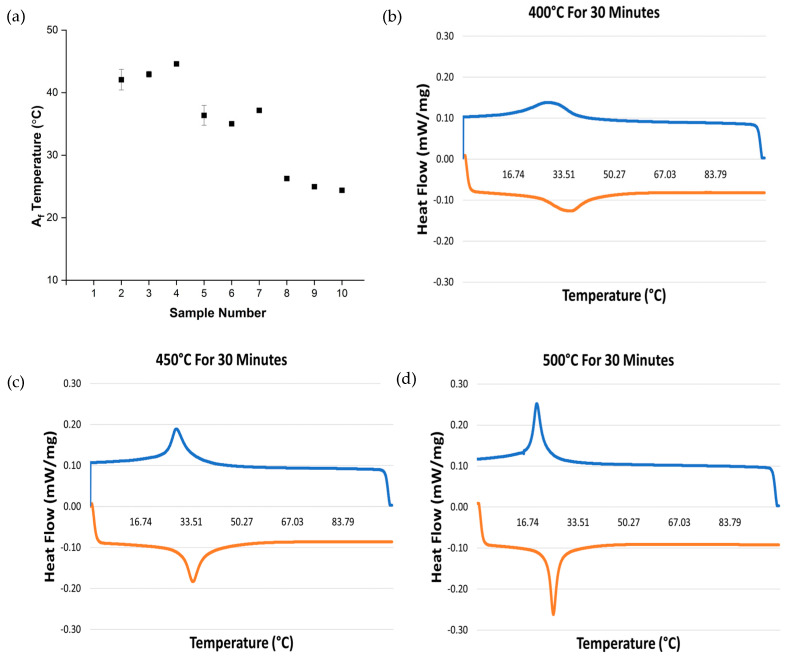
(**a**) The A_f_ phase transformation temperatures of the wire samples (*n* = 2), and DSC heating curves (orange line) and cooling curves (blue lines) of samples treated (**b**) at 400 °C for 30 min, (**c**) at 450 °C for 30 min, and (**d**) at 500 °C for 30 min.

The phase change, including the austenite finish, temperatures (A_f_) were measured using DSC, see Figure 6. These results show that there was a decrease in the A_f_ temperatures with increasing heat treatment temperature. The lowest A_f_ temperature for a specific temperature group was higher than the highest A_f_ temperature found in the next lower temperature treated group. On the other hand, there was an increase in A_f_ temperature with time for samples heated at 400 °C, a decrease in A_f_ temperature with time at 500 °C, and relatively little effect of time at 450 °C on the A_f_ temperature. From the DSC curves, the phase change peaks become sharper with both heat treatment temperature and time with the broadest peak at 400 °C for 30 min and the sharpest at 500 °C for 120 min.

### 3.4. Effect on Hardness Due to the Heat Treatment Process

**Figure 7:**
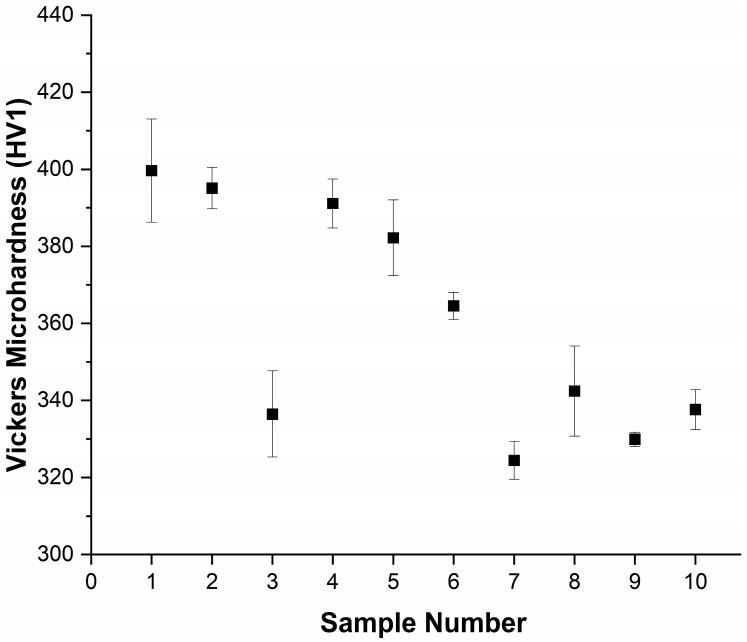
Vickers microhardness test results for as-received and heat-treated samples, *n* = 5.

Figure 7 presents the results from the Vickers microhardness indentation tests. The measured hardness for all the heat-treated samples in this study were found to be lower than the measured hardness of the as-received nitinol wire. For the samples treated at 400 °C (samples 2, 3, and 4), there was an initial decrease in the hardness values between the samples treated at 30 min (sample 2) and those at 60 min (sample 3), and a subsequent increase in the hardness values from the 60 min samples (sample 3) to the 120 min (sample 4) heat-treated samples. For the samples treated at 450 °C (samples 5, 6, and 7), there was a general trend that showed a decrease in the hardness of the samples as the heat treatment time increased [30,37]. Increasing heat treatment temperature also resulted in a reduction in average hardness. The trend of results of this work in terms of hardness matched well with those recorded previously [38].

### 3.5. Loss of Actuation Angle for the Nitinol Wires

**Figure 8:**
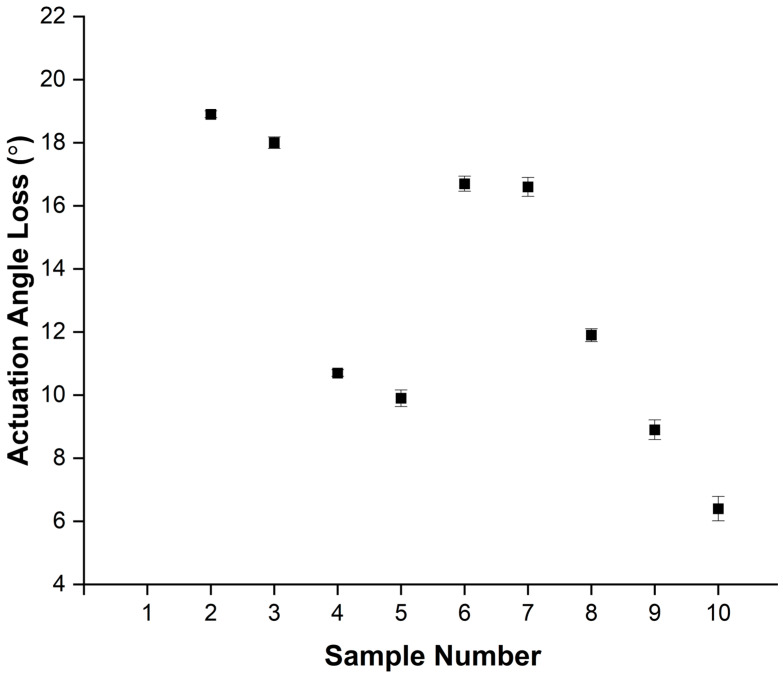
Loss of actuation angle from desired shape (°) for the heat-treated samples.

The results in Figure 8 show that the largest deviations from the straight wire occurred in samples heat treated at 400 °C for 30 (sample 2) and 60 min (sample 3) with angles of 18.80° and 18.16° degrees, respectively. Increasing the heat treatment time to 120 min at 400 °C resulted in a reduction of this angle to 10.61°.

Samples heat treated at 450 °C show a different relationship between angle and heat treatment time. The lowest angle occurred in the sample heated for 30 min (sample 5), achieving an angle of 10.10°. The angle increased drastically to 16.92° at 60 min (sample 6) and showed only a slight decrease to 16.74° for 120 min (sample 7).

For samples heat treated at 500 °C, a decrease in angle with increasing heat treatment time was observed. The sample treated for 30 min (sample 8) achieved an angle of 12.07°. The samples treated at 60 min (sample 9) and 120 min (sample 10) both produced the lowest actuation angular loss compared to any of the other samples at 9.22° and 6.75°, respectively, as shown in Figure 8.

### 3.6. Recovery Strain of the Heat-Treated Nitinol Wires

**Figure 9:**
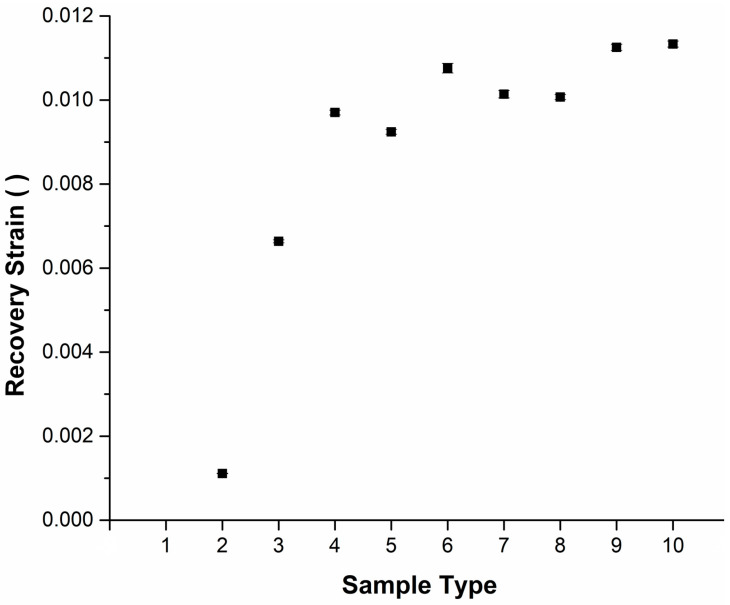
Recovery strain values for the heat-treated samples, *n* = 3.

The recorded recovery strains of the heat-treated nitinol wires, after bending the wires and then recovering once they were heated above the A_f_ temperature, are shown in Figure 9. For the samples heat treated at 400 °C, an increasing extent of recovery in the strain values was observed with an increasing heat treatment time. A similar trend was observed for the samples heat treated at 500 °C with maximum recovery strain achieved for the sample heat treated at 500 °C for the longest duration of 120 min.

For the samples heat treated at 450 °C, the lowest time of 30 min showed the lowest recovery in the strain values. Increasing the time of 60 min heat treatment showed an increase in the recovery strain value, whereas a further increase in the heat treatment time to 120 min resulted in a drop in the recovery strain value.

## 4. Discussion

Main aims of this study were to determine how the nitinol wire heat treatment process parameters relate to the resulting shape recovery response and mechanical properties. In this work, wire samples were heat treated in a box furnace at three different temperatures of 400 °C, 450 °C, and 500 °C with varying times of 30, 60, and 120 min for each temperature. The samples were heat treated in atmospheric conditions and air cooled outside the furnace. As shown in Figure 9, the recovery strain response of the heat-treated samples increased as the heat treatment temperature increased. A maximum recovery strain value was observed for the wires heat treated at 500 °C for 120 min. This result correlates well with the measured loss of actuation angle that was found to be the lowest for the wires heat treated in this condition, see Figure 8. There was an increase in the change in the radius of curvature of the wire as the heat treatment temperature of the wire was increased. Wires with the highest recoverable strain provide the maximum shape recovery capability.

The EDX analysis of the post-processed wires showed no significant changes in the chemical composition of the samples. The chemical composition of nitinol significantly influences the performance of the shape memory alloy, demanding close monitoring of the sample’s composition. As mentioned earlier, the chemical composition of the samples used in this study was found to be Ni55 wt.% and Ti45 wt.%. A more Ti-rich nitinol alloy exhibits better shape memory property and higher phase transformation temperature [39,40].

The density of the phases present in the produced parts can be directly correlated to the hardness values measured. Ni_4_Ti_3_ precipitates have been identified in heat-treated NiTi samples with more precipitates occurring at higher aging temperatures and with longer heat treatment periods as reported by Rondelli et al. [30]. The lower hardness values for the samples heat treated at 450 °C and 500 °C is likely caused by the increased presence of the Ni_4_Ti_3_ precipitates in the wire samples, thereby lowering the hardness values compared to samples heat treated at 400 °C [38,41]. As indicated previously in the study conducted by Khalil-allafi et al. [16], at the ageing temperature range of 400 to 600 °C, heterogenous distribution of the Ni_4_Ti_3_ precipitates has been observed. It has also been reported that the grain size increases with increasing ageing temperature that leads to lowering of the hardness values. In the work of Adharapurapu et al. [38], the samples that were heat treated at 400 °C for 5 h exhibited the best hardness values. From this previous study, the sample heat treated at 500 °C for 5 h minutes also displayed a lower hardness value, however, for sample heat treated at this temperature for longer periods of 10 h or more, the hardness increased. This finding directly correlates with our results. The trend of decreasing hardness values with increasing temperatures and times also is expected to be attributed to the formation of an increased Ti-rich matrix due to the formation and growth of Ni_4_Ti_3_ and Ni_3_Ti_2_ precipitates.

A clear indication of the changes in the A_f_ temperature has been observed because of the heat treatment process, see Figure 6. From the graph, it is apparent that there is a decrease in A_f_ temperature with increasing heat treatment temperature. The heat treatment time, however, has a relatively small effect on the transformation temperatures, indicating that the main driver for the reduction in A_f_ is the heat treatment temperature rather than time. The samples heat treated at same temperature of 400 °C but for varying times, however, show a general increase in A_f_ temperature with heat treatment time that has also been reported in the literature due to an increase in Ni_4_Ti_3_ precipitation [42,43]. Furthermore, a noticeable increase in the sharpness of the transformation peaks was observed with extended time and temperature. This trend was accompanied by a decrease in A_f_ and a heightened precision in the transformation peaks, as both temperature and time increased [17,44]. This phenomenon can be attributed to the relaxation of residual stress and the correction of microstructural defects that hinder crystal mobility during heating. The alteration in the A_f_ temperature caused by the heat treatment process is advantageous, as it offers the potential for adjusting the phase change temperature according to specific application requirements.

In this work, a straightforward and quick method of calculating the actuation of a wire at the actuation angle was developed. The results show that the actuation improved with both heat treatment temperature and time. The largest shape recovery was achieved with the sample heated at the highest temperature for the longest time (500 °C for 120 min), while the lowest shape recovery was recorded at the lowest temperature and shortest time (400 °C for 30 min). The growth of the metastable finer Ti-rich precipitates with the increase in the heat treatment temperature to 500 °C resulted in more martensitic phases that increased the shape memory response.

This study explores the impact of heat treatment parameters on the performance of nitinol wires, specifically focusing on actuation characteristics. While the primary focus is on understanding the material behavior, the results of this research can have direct applicability in industries where nitinol-based actuators are used. By optimizing the heat treatment parameters, the study shows how to enhance the actuator performance, which is valuable for the development of robotic, aerospace, and medical actuator devices. This work, therefore, adds a new scientific knowledge base to inform future practical product designs. The study utilizes a novel approach involving image analysis to measure recovery strain during wire actuation. This methodological innovation can be used by the scientific community as a new practical and robust technique for assessing shape memory alloy behavior.

## 5. Conclusions

Wire samples of nitinol manufactured using traditional wire-drawing techniques were examined to determine the effect of heat treatment time and temperature on the density, hardness, material composition, surface morphology, transformation temperature, angular displacement, and strain recovery. This study highlights that the homogeneity and presence of precipitates of nitinol are critical to final properties of the material. The conclusions drawn from the obtained results are as follows:

1. There is a decrease in the density between the as-received samples and the heat-treated samples, except for sample 3. Sample 3 was treated at 400 °C for 60 min and an increase from the as-received density of 6.45 g/cm^3^ to 6.85 g/cm^3^ was observed. The results show that the heat treatment time and temperature can be used to control the density within the range of 5.85 to 6.85 g/cm^3^.
2. The hardness results show a general decrease in hardness with heat treatment time and temperature.
3. From the EDX and SEM results, the as-received wire has a uniform distribution of Ni and Ti with a surface oxide layer due to titanium’s highly oxidizing nature.
4. The DSC curves show that increasing heat treatment time and temperature decreases the A_f_ temperature and increases the sharpness of the phase transition curves. This indicates that higher temperatures (500 °C) for longer times (120 min) results in an increased reduction in residual stresses and defects that impede crystal mobility.
5. NiTi heat treated at 400 °C for up to 60 min and 450 °C for 60 to 120 min fails to result in a high level of shape memory response. Heat treating NiTi at 500 °C for 60 (8.9°) and 120 min (6.4°) results in an increasing shape memory effect with the greatest shape recovery observed in the sample treated for 120 min.
6. The strain recovery increases with increasing heat treatment time and temperature. The maximum strain recovery occurred in the sample heated at 500 °C for 120 min.

In terms of limitations, the size of the samples was too small to allow XRD testing. Sheet samples could potentially be used to allow for XRD analysis due to its larger surface area. Future studies should prioritize in-depth investigations aimed at achieving the maximum shape memory effect tailored specifically to the desired application. Future work could also examine the heat treatment of the wire samples at higher temperatures (e.g., 550 °C or 600 °C) and for longer periods of time (e.g., 150 and 180 min). More rapid cooling of the samples such as the use of water quenching of the wires after the heat treatment process should also be further examined.

## Figures and Tables

**Table 1:** Heat treatment process parameters applied to the nitinol wires.

Sample Number	Temperature (°C)	Time (min)
1	As-received	As-received
2	400	30
3	400	60
4	400	120
5	450	30
6	450	60
7	450	120
8	500	30
9	500	60
10	500	120

**Table 2:** Chemical composition of as-received and heat-treated nitinol wires.

Sample Number	Ni (at.%)	Ti (at.%)
1	49.9	50.1
2	49.3	50.7
3	49.5	50.5
4	49.4	50.6
5	47.9	52.1
6	48.8	51.2
7	49.1	50.9
8	48	52
9	47.5	52.5
10	46	54

## Data Availability

Data will be made available on request.

## References

[1] Sadrnezhaad K., Mashhadi F., Sharghi R. (1997). Heat Treatment of Ni-Ti Alloy for Improvement of Shape Memory Effect. Mater. Manuf. Process..

[2] Elahinia M.H. (2016). Shape Memory Alloy Actuators: Design, Fabrication, and Experimental Evaluation.

[3] Mao C., Dong J., Li H., Ou J., Tomizuka M., Yun C.-B., Lynch J.P. (2012). Seismic Performance of RC Shear Wall Structure with Novel Shape Memory Alloy Dampers in Coupling Beams.

[4] Fu C.H., Sealy M.P., Guo Y.B., Wei X.T. (2014). Austenite-Martensite Phase Transformation of Biomedical Nitinol by Ball Burnishing. J. Mater. Process. Technol..

[5] Guo Y., Klink A., Fu C., Snyder J. (2013). Machinability and surface integrity of Nitinol shape memory alloy. CIRP Ann..

[6] Liu X., Wang Y., Yang D., Qi M. (2008). The Effect of Ageing Treatment on Shape-Setting and Superelasticity of a Nitinol Stent. Mater. Charact..

[7] Oncel L., Acma M.E. (2017). Effect of Heat Treatment Temperature and Heat Treatment Time on Properties and Use of NiTi Shape Memory Implant Material. IARJSET.

[8] Vojtěch D. (2010). Influence of Heat Treatment of Shape Memory Niti Alloy on Its Mechanical Properties. Metal.

[9] Metals F.W. Shape Setting. https://www.fwmetals.com/materials/nitinol/nitinol-shapesetting/.

[10] Benafan O., Brown J., Calkins F.T., Kumar P., Stebner A.P., Turner T.L., Vaidyanathan R., Webster J., Young M.L. (2014). Shape Memory Alloy Actuator Design: CASMART Collaborative Best Practices and Case Studies. Int. J. Mech. Mater. Des..

[11] Pelton A.R., Russell S.M., Dicello J. (2003). The Physical Metallurgy of Nitinol for Medical Applications. JOM Phys. Metall..

[12] Drexel M., Selvaduray G., Pelton A. The Effects of Cold Work and Heat Treatment on the Properties of Nitinol Wire. Proceedings of the Medical Device Materials IV: Materials and Processes for Medical Devices Conference 2007.

[13] Kök M., Dağdelen F., Aydoğdu A., Aydogdu Y. (2016). The Change of Transformation Temperature on NiTi Shape Memory Alloy by Pressure and Thermal Ageing. Proceedings of the Journal of Physics: Conference Series.

[14] Otsuka K., Ren X. (2005). Physical Metallurgy of Ti-Ni-Based Shape Memory Alloys. Prog. Mater. Sci..

[15] (1987). Ageing. Ann. Acad. Med. Singapore.

[16] Khalil-Allafi J., Dlouhy A., Eggeler G. (2002). Ni_4_Ti_3_-Precipitation during Aging of NiTi Shape Memory Alloys and Its Influence on Martensitic Phase Transformations. Acta Mater..

[17] Sadiq H., Wong M.B., Al-Mahaidi R., Zhao X.L. (2010). The Effects of Heat Treatment on the Recovery Stresses of Shape Memory Alloys. Smart Mater. Struct..

[18] Ma X., Zhou Y., Chen X., Lu G., Ji J., Zhao S. (2021). Effect of Temperature on Phase Transformation of NiTi-Based Shape Memory Alloy. J. Phys. Conf. Ser..

[19] Golberg D., Xu Y., Murakami Y., Otsuka K., Ueki T., Horikawa H. (1995). High-Temperature Shape Memory Effect in Ti_50_Pd_50-X_Ni_x_ (x = 10, 15, 20) Alloys. Mater. Lett..

[20] Lahoz R., Puértolas J.A. (2004). Training and Two-Way Shape Memory in NiTi Alloys: Influence on Thermal Parameters. J. Alloys Compd..

[21] Luo H.Y., Abel E.W. (2007). A Comparison of Methods for the Training of NiTi Two-Way Shape Memory Alloy. Smart Mater. Struct..

[22] Speicher M., Hodgson D.E., Desroches R., Leon R.T. (2009). Shape Memory Alloy Tension/Compression Device for Seismic Retrofit of Buildings. J. Mater. Eng. Perform..

[23] Aryan H., Ghassemieh M. (2017). A Superelastic Protective Technique for Mitigating the Effects of Vertical and Horizontal Seismic Excitations on Highway Bridges. J. Intell. Mater. Syst. Struct..

[24] Semba H., Okabe N., Yamaji T., Okita K., Yamauchi K. (2005). Axial Compressive Behavior of Single-Stage Bellows of TiNi Shape Memory Alloy for Seismic Applications. Mater. Sci. Forum.

[25] Reedlunn B., Daly S., Shaw J. (2013). Superelastic Shape Memory Alloy Cables: Part i-Isothermal Tension Experiments. Int. J. Solids Struct..

[26] Shaw J.A., Grummon D.S., Foltz J. (2007). Superelastic NiTi Honeycombs: Fabrication and Experiments. Smart Mater. Struct..

[27] Taha O.M.A., Bahrom M.B., Taha O., Aris M.S. (2015). Experimental study on two way shape memory effect training procedure for NiTiNOL shape memory alloy. ARPN J. Eng. Appl. Sci..

[28] (2008). Mechanical Errors. Precis. Manuf..

[29] (2013). NaberTherm NaberTherm Laboratory Furnaces Brochure 2021.

[30] Rondelli G., Vicentini B. (1999). Localized Corrosion Behaviour in Simulated Human Body Fluids of Commercial Ni-Ti Orthodontic Wires. Biomaterials.

[31] Labratories E. (2017). Density Determination of Solids and Liquids 2021. ASTM B962: Standard Test Methods for Density of Compacted or Sintered Powder Metallurgy (PM) Products Using Archimedes’ Principle.

[32] Elmer P. Guide to Selection of Differential Scanning Calorimetry (Dsc) Sample Pans 2021. https://resources.perkinelmer.com/lab-solutions/resources/docs/tch_guide-to-dsc-selection-pans.pdf.

[33] Commons W. User A1 2021. https://www.hardnessgauge.com/testing-types/vickers-hardness-testing/.

[34] Rueden C.T., Schindelin J., Hiner M.C., DeZonia B.E., Walter A.E., Arena E.T., Eliceiri K.W. (2017). ImageJ2: ImageJ for the next Generation of Scientific Image Data. BMC Bioinform..

[35] Buehler W.J., Wang F.E. (1968). A Summary of Recent Research on the Nitinol Alloys and Their Potential Application in Ocean Engineering. Ocean Eng..

[36] Xu L., Wang R. (2010). The Effect of Annealing and Cold-Drawing on the Super-Elasticity of the Ni-Ti Shape Memory Alloy Wire. Mod. Appl. Sci..

[37] Lee J., Shin Y.C. (2019). Effects of Composition and Post Heat Treatment on Shape Memory Characteristics and Mechanical Properties for Laser Direct Deposited Nitinol. Lasers Manuf. Mater. Process..

[38] Adharapurapu R.R., Jiang F., Vecchio K.S. (2010). Aging Effects on Hardness and Dynamic Compressive Behavior of Ti-55Ni (at.%) Alloy. Mater. Sci. Eng. A.

[39] Haberland C., Elahinia M., Walker J.M., Meier H., Frenzel J. (2014). On the Development of High Quality NiTi Shape Memory and Pseudoelastic Parts by Additive Manufacturing. Smart Mater. Struct..

[40] Haberland C., Meier H., Frenzel J. SMASIS2012-8040 On the Properties of Ni-Rich Niti Shape Memory Parts Produced by Selective Laser Melting. Proceedings of the ASME 2012 Conference on Smart Materials, Adaptive Structures and Intelligent Systems.

[41] Stockel D. (1998). Endovascular Update 1.1.

[42] Gallardo Fuentes J.M., Gumpel P. (2002). Phase Change Behavior of Nitinol Shape Memory Alloys. Adv. Eng. Mater..

[43] Spini T.S., Valarelli F.P., Cançado R.H., Freitas K.M.S.d., Villarinho D.J. (2014). Transition Temperature Range of Thermally Activated Nickel-Titanium Archwires. J. Appl. Oral Sci..

[44] Marattukalam J.J., Balla V.K., Das M., Bontha S., Kalpathy S.K. (2018). Effect of Heat Treatment on Microstructure, Corrosion, and Shape Memory Characteristics of Laser Deposited NiTi Alloy. J. Alloys Compd..

